# Developing an educational scheme for undergraduate medical Curriculum: the unit of "INFERTILITY" as a sample

**Published:** 2012-03-05

**Authors:** A Aflatoonian, B Baghianimoghadam, A Abdoli, P Partovi, P Hemmati, N Tabibnejad, P Harasym

**Affiliations:** * Research and Clinical Center for Infertility, Shahid Sadoughi University of Medical Sciences, Yazd, Iran; ** Center for Disease Control, Deputy Ministry for Health Affairs, Ministry of Health and Medical Education, Tehran, Iran; *** Faculty of Medicine, University of Calgary, Alberta, Canada

**Keywords:** CPC, scheme, infertility, backward reasoning, forward reasoning

## Abstract

**Objectives: **to present our first experience in scheme development based on CPC philosophy in Iran.

**Hypothesis: **One of the most important reasons of an obvious gap between medical education and professional expectations (outcomes) encountered by recent medical graduates is due to applying conventional curricula, which rely on hypothetical-deductive reasoning model. The University of Calgary has implemented a new curriculum which is organized according to 125 ways in which patients may present to a physician. In this study we will present our first experience in scheme development based on CPC philosophy in Iran.

**Methods: **In 2007, research and clinical center for infertility (Yazd University of medical sciences, IRAN), began developing a full module for infertility (lesson plan) with fourteen components based on the new curricular philosophy. We recruited a scheme of infertility according to a specific way.

**Results: **Thus, at the first step of the module creation, a scheme was made as the most important mainstay of presentation module, i.e. a structured scheme that includes all causative diseases of infertility.

**Conclusions: **Any effort in the organization of knowledge around schemes including in the domain of infertility would be valuable to meet some of the standards of WFME. Also, development of modules, by the teams composed of experts and students, can improve the quality of medical education.

## Introduction

Medical education and medical systems expectations are different, medical graduates encounter some problems that the educational curriculums did not supply [**1-3**]. One reason may be routine conventional curricula, which rely on hypothetical-deductive reasoning model (HDR or backward reasoning or disease-centered medical education) [**4,5**]. This philosophy returns to Dr. Abraham Flexner’s ideas, who made the difference between the hypothetical-deductive reasoning model from the model of reasoning in basic sciences and applied it for medical education too [**6**]. In fact, the influence and strength of Flexner in his era made a lot of his concurrent educators to follow his idea. Therefore, the effect of that idea remained up to now even in modern Problem Based Learning (PBL) curricula [**6**].

The patient did not come with a name of his disease; the patient has not read medical books!! In fact, he talks about his complaint (or clinical presentation for example chest pain, cough, dysuria. etc.). The current educational direction is from disease to manifestations. Supporters of this type of reasoning believe that there is a serious need for a hypothesis generated beforehand in order to enable one to set an inquiry strategy. But, this type of problem-solving, may not be completely appropriate for problem solving under the constraints of clinical setting (i.e. constraints of time, knowledge and skills) [**5,7**], especially considering that this direction practices a deliberate trial-and-error approach on a human subject, being unethical. Barrows and Pickell proposed a backward clinical problem solving model [**4**]. In this model and also PBL (that has borrowed its philosophy from problem solving), the nature of traditional educational curricula is apparent. Even in PBL that starts from case presentation (Problem), as it name implies, in the interim of its process, it somehow returns to disease-centered education. Accordingly, we can see Flexner’s view of hypothetico-deductive reasoning. But, this cannot support the needs of clinic. However, if it comes to developing the quality of the medical education, we must pull out the way of reasoning that is going on in the expert’s mind [**8,9**]. Actually, because of his experience, an expert can go from clinical presentation to diseases; and this is the way we want it to happen. In fact, an expert has a broad picture of all suspected etiologies of clinical presentation and discriminates them together with the key predictors. 

In an attempt to advance the quality education for medical students, the University of Calgary medical school implemented a new curriculum in the fall of 1994. The previous systems-based curriculum was reorganized according to 120 clinical presentations (CPs) or problem domains. The CP model has been described in detail elsewhere [**10**]. A clinical presentation is defined as a common and important way in which patients present to a physician. Examples of clinical presentations include headache, abdominal pain, sore throat, and hypertension. Another unique feature of the CP curriculum is the introduction of 'scheme' to students contained in the terminal objectives of each clinical presentation.

Regarding the problems arisen by conventional curricula and defined by Haeri, Hemmati, and Yaman [**11**], we decided to develop the first module according to the last curricular model in the North America, i.e. Clinical Presentation Curriculum (CPC). The aim of this study was to present the first experience in scheme development, based on CPC philosophy in Iran.

## Methods

Infertility is defined as the inability of a couple to conceive after 12 months of regular, unprotected intercourse. Infertility is a popular presentation in the gynecology and urology field. Due to the complexity of its causes female infertility has a very complicated diagnosis. However, a GP as the first line in diagnosis and management must be capable of categorizing the causes of this presentation. 

The University of Calgary has implemented a new curriculum, which is organized according to 120 ways in which patients may present to a physician.

Each module will contain 14 components: the logical development of a scheme, an expert’s scheme, matrix, terminal objectives, enabling objectives, basic science content list, schedule, teaching materials (i.e. PowerPoint slides), learning materials (i.e. reading assignments), PBL cases, process worksheet for tutors to guide small group PBL scheme-inductive sessions, formative evaluations, summative evaluations, and remedial intervention.

In 2007, the research and clinical center for infertility (Yazd University of medical sciences, IRAN), began developing a full module for infertility (lesson plan) with fourteen components based on clinical presentation curricular philosophy. This program was as a part of making 10 packages ordered by the Ministry of Health and medical education jointed by WHO.

At first, we recruited a research team, combined of four medical students chosen among talented researchers, a professor (clinical expert) in infertility and medical educationalist. During the study phase we have also benefited from consultations from the other members of Gynecology and Infertility Department, Faculty of Medicine at Yazd University of Medical Sciences.

During the study phase of generating the material of curriculum, we received guidelines. In the first step, our medical educationalist gave us three structured series of questions. With the aim of those questions, we gathered detailed information about the presentation and the prototypical diseases that our expert chose, those that are the most important diseases that a GP must know and can manage completely.

So, we had to look for the most popular definition of infertility. Fortunately, all the textbooks have a common and comprehensive definition mentioned above. 

**Fig. 1:**
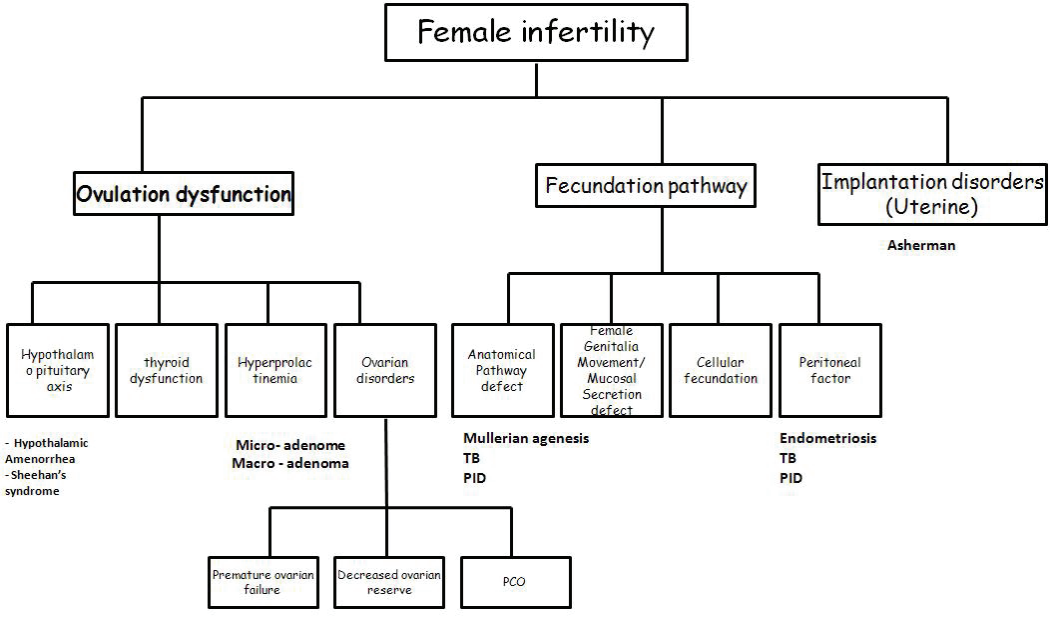
Infertility scheme finalized in research and clinical center for infertility, Yazd, Iran

We started from a watchful study on basic physiology and anatomy of female reproductive tracts. Then, we searched for all diseases that can cause infertility and we tried to extract the mechanism of the disease in creating the infertility. According to similarities in the anatomic and/or physiologic aspects of causative diseases of infertility, we made a primary classification of all possible disorders. We gathered all diseases with the same mechanism into one “disease class”. This was the bottom of our scheme. Then based on a mechanism we tried to select a meaningful name for this group of diseases, as the smallest unit of our classification (this smallest units at the bottom of the schemes are called “disease class” in CP curriculum). Then, based on similarities, we tried to compile a few disease classes into one subcategory (or sub subcategory) and continuing this strategy we made greater sets of assembles until achieving the main categories in the first layer of the scheme (ovulation dysfunction, fecundation pathway and implantation disorders), then to clinical presentation itself (infertility) (a bottom – up scheme construction). (See **Fig. 1** in the Results section).

All the process of this study was evaluated by experts and a medical educationalist of our group every weekend!! At any session, we got our expert comment on our scheme and he compared our plan with the road map in his mind (that was our aim to pull it out of his mind) and checked every entity (category/subcategory/disease class/disease differentials) for the existence of diagnostic key predictors. If a key predictor/s exists he approved the entity. The study group reported their studies in Power Point Presentations in every meeting. In fact, the above scheme was the fruit of studying around the first series of questions. 

Thus, the clinician has identified the differentials of the clinical presentations (CP) in one’s field of expertise. For each CP he/she has organized the differentials based on common attributes (i.e., anatomy, and physiology) into categories, subcategories, disease classes and short lists of cohort differentials in each class. Such a knowledge structure is called a scheme. And with its demonstration, medical students would get to a more organized knowledge structure in a shorter period.

After developing the matrix of infertility, we continued to gather all information about prototypical diseases, according to the second and third series of structured questions, to complete the learning material component of a CP module.

## Results

The scheme created by the study group is offered in **Fig. 1**.

In our experience in developing infertility package based on CPC model, as mentioned in materials and methods, we have made a scheme about female infertility that included all diseases causing this CP. We divided female infertility into three main causes (category): ovulation dysfunction, fecundation pathway and implantation disorders (uterine). The fecundation pathway is a new term that we devised to make the first line of our scheme at the same level with the other two main categories. The fecundation pathway represents the way that sperm must transfer from external genitalia to ovum and fecundate. Ovulation dysfunction is divided to four sub categories: hypothalamopituitary axis disorders, thyroid disorders, hyper prolactinemia and ovarian disorders. Ovarian disorders divided into polycystic ovarian syndrome, premature ovarian failure and decreased ovarian reserve. The fecundation pathway is divided into anatomical pathway defects, female genitalia/mucosal secretion defects, cellular fecundation and peritoneal factors. The implantation disorders do not have a sub category.

Making this scheme took a long time, as our first experience, because the generation of every surface of scheme was like a workshop for us. The generation of schemes needed a careful definition of CP because all the diseases that must be included in scheme, in first step, must have our definition specification. And then, we needed that part of the disease that makes our CP. For example, TB as an infectious disease makes a large variety of presentations with multiple organ involvement. In fact, only genital TB is important for us; other types of TB with theirs specifications do not include our scheme.

## Discussions

Up to now all the medical education curricula were based on a hypothetical-deductive reasoning model.

Now, mankind experience in education, benefiting from cognitive sciences, has helped to develop a new curriculum that is concomitant with the nature of disease. Each disease starts from a disturbance in a normal state of the body. Then, a pathology that leads to a specific sign or a symptom in a patient appeared and made the Clinical Presentation (CP)! A Patient talks about this CP not the name of his disease!! In the diagnosis of the disease an expert does not use the hypothetical deductive reasoning; in fact, after a long time of experiences we learnt to reorganize his knowledge (that primarily learnt based on hypothetical deductive reasoning) around the natural state, that a patient comes with (clinical presentation).

The new curriculum, designed and implemented in the University of Calgary, is based on this natural necessity of clinical setting. Moreover, we tried to develop the first samples of CP modules in this curriculum, in Iran. The development in the quality of the medical education implemented with each new curriculum birth. Each new model used other older curriculum positive aspects and tried to decrease their weaknesses. CPC (clinical presentation curriculum) is the latest invention in the curriculum development that gives a new implementation with scheme, CP matrix and learning objectives.

With these discussions, CPC does not render other previous curricula such as Problem Based Learning (PBL) and Organ System Based Curriculum (OSBC). In fact, in this curriculum, the philosophy of education based on presentation (the main trait of PBL) and also the structure of system by system education (the main trait of OSBC) is saved, but this curriculum tries to solve the inadequacies in other curriculums and make a physician with empowerment in differential diagnosis and making a correct diagnosis in shortest time. 

Schemes have three major advantages:

1- They make students capable to categorize their information in mind because in this method we prune all unnecessary information that may interfere the processing of information in mind.

2- The schemes develop a scaffold in students’ mind, which help them to connect all relevant information especially basic science issues. In our experience, we used an innovative method to integrate the infertility scheme with the basic science concepts that help student to deeply understand the puzzle (our scheme) and memorize the scheme more scientifically as well as their previously learnt information in basic sciences

3- The schemes offer a clinical problem-solving strategy which start from the CP toward disease differentials (forward reasoning strategy), which is different with the hypothetical-deductive (backward) reasoning strategy (bottom-up diagnosis) offered by Barrows et al. The forward reasoning is more effective than backward reasoning in regard to the speed and accuracy of clinical diagnosis. This phenomenon has been shown by Coderre et al.

In a research implemented in 1991 by Norman and Patel [**12**] results showed that student graduated in department based curriculum were better in forward reasoning in contrast with student graduated in PBL model!! Students in PBL model only could do Backward reasoning! In justification of this incredible phenomenon authors said that frequent use of backward reasoning in PBL and use of Non Expert Tutors are the causes.

In other research that implemented by Mandin and Harasym in 1997 [**13**], researchers understood that integration of clinical and basic science information by CPC model redounded to reinforcement of student in diagnosis process (in contrast with other old models that believed usage of clinic and basic science separately). 

In another research implemented by Woloschuk, Harasym, Mandin & Jones in 2007 [**14**] on education based on CPC on medical students in university of Calgary, they resulted that the student response to schemes has been favorable. 

All this results shows that more and more effort in organization and regularization of lesson plan and its component can help us in gaining better and better outcomes. Then it’s clear that efforts of this team in organization of infertility package is valuable because module development by various teams and experts can improve quality of medical education. Also development of modules by the teams composed of experts and students can improve quality of medical education.
